# A143 PREDICTIVE SCORES AND CLINICAL FAILURE AFTER PER-ORAL ENDOSCOPIC MYOTOMY

**DOI:** 10.1093/jcag/gwae059.143

**Published:** 2025-02-10

**Authors:** M K Parvizian, M Rai, R Bechara

**Affiliations:** Queen’s University, Kingston, ON, Canada; Queen’s University, Kingston, ON, Canada; Queen’s University, Kingston, ON, Canada

## Abstract

**Background:**

Achalasia is a rare esophageal motility disorder characterized by incomplete lower esophageal sphincter relaxation and absent peristalsis. Increasingly, per-oral endoscopic myotomy (POEM) has been used as a first-line therapy for achalasia. However, a small proportion of patients will experience clinical failure after POEM (Eckhardt Score>3). Several small studies have attempted to predict which patients will experience failure after POEM; however, none have been validated outside their original centers.

**Aims:**

To compare the performance of the Zhongshan, JAMS, and Urakami scores to predict key outcomes post-POEM.

**Methods:**

A single centre retrospective cohort study from March 2016 to January 2024 including all adult patients undergoing POEM for achalasia with at least 3 months of follow-up was conducted. The performance of the different scores in predicting clinical failure and the presence of minimal symptoms (Ekhardt Score <=1) was analyzed. Scores were calculated and patients categorized as high-risk for failure (>=5% risk) or low risk based on score cutoffs from the original validation studies.

**Results:**

156 patients were included in our study. The median age of participants was 57.5 (IQR 36-68), and 70 were female (44.9%). 6 patients experienced clinical failure (3.8%) and 115 had minimal symptoms post-POEM (73.7%). The Urakami score demonstrated the highest sensitivity for predicting clinical failure (1.00; 95% CI 0.54-1.00) and presence of more than minimal symptoms (1.00; 95% CI 0.91-1.00), while the Zhongshan score exhibited the highest specificity for clinical failure (0.82; 95% CI 0.74-0.88) and more than minimal symptoms (0.84; 95% CI 0.76-0.90). (**Table 1**)

**Conclusions:**

This study reports the first validation of predictive scores in POEM patients outside of their centres of origin. The Urakami score may play a role in identifying patients at low risk of clinical failure, while the Zhongshan score may help identify patients at high risk of clinical failure. Using these scores can aid in counseling patients pre-POEM about their expected outcomes.

Test characteristics of various scores to predict outcomes after POEM

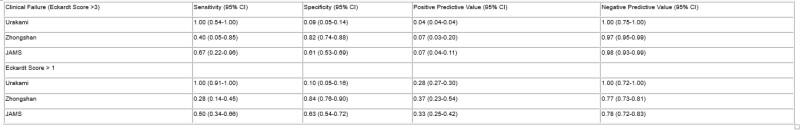

**Funding Agencies:**

None

